# Supplementary material for: Taxonomic and environmental distribution of bacterial amino acid auxotrophies
Source: Nat Commun. 2023 Nov 22;14:7608. doi: 10.1038/s41467-023-43435-4 (PMC10665431; doi:10.1038/s41467-023-43435-4)
Supplement: Supplementary file 1 — Supplementary Information [file 41467_2023_43435_MOESM1_ESM.pdf]

Supplementary Information

Supplementary Figure 1. Validation of the predictive model of amino acid auxotrophy using taxa that grow in minimal media. Predictive model accuracy across 171 bacterial taxa that grow in minimal media (prototrophic taxa) considering a taxon to be auxotrophic for a given amino acid when the model could not find a confident match for at least 20% (A), 30% (B), 40% (C), or 50% (D) of the genes belonging to that given amino acid biosynthesis pathway in the representative genome.

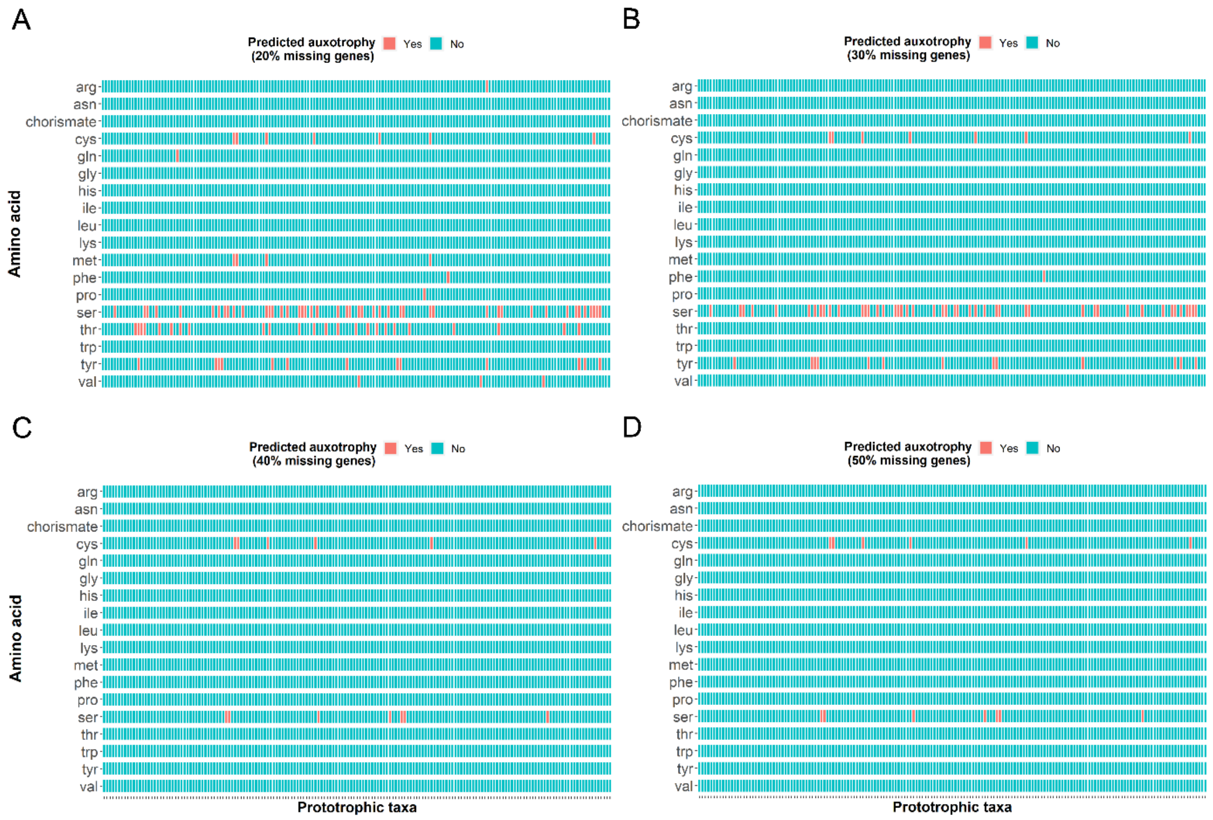

Supplementary Figure 2. Validation of the predictive model of amino acid auxotrophy using taxa with experimentally determined amino acid auxotrophies. Predictive model accuracy for 19 taxa with experimentally determined amino acid auxotrophies considering a taxon to be auxotrophic for a given amino acid when the model could not find a confident match for at least 20% (A), 30% (B), 40% (C), or 50% (D) of the genes belonging to that given amino acid biosynthesis pathway in the representative genome. Tiles colored in black indicate there was no experimental verification of the biosynthesis capabilities of those taxa for those amino acids.

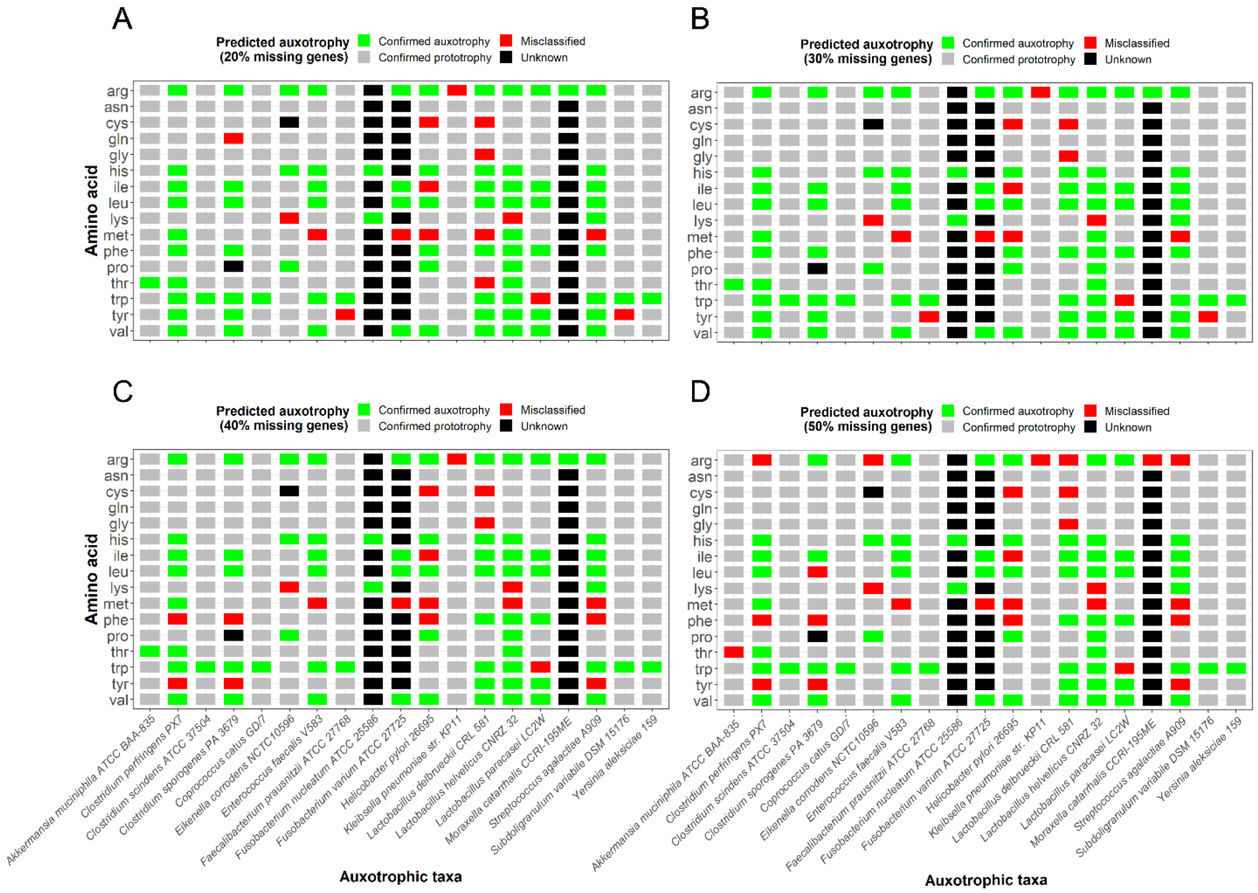

Supplementary Figure 3. Summary of amino acid auxotrophy across the predominant bacterial families. The heatmap depicts the proportion of taxa that are auxotrophic for a given amino acid out of all auxotrophic taxa within that family. Column “N” shows the total number of sequenced genomes for which we predicted amino acid auxotrophy in each family. We included all families belonging to the predominant bacterial phyla that had more than 100 sequenced genomes with more than 95% completeness in the Genome Taxonomy Database (GTDB).

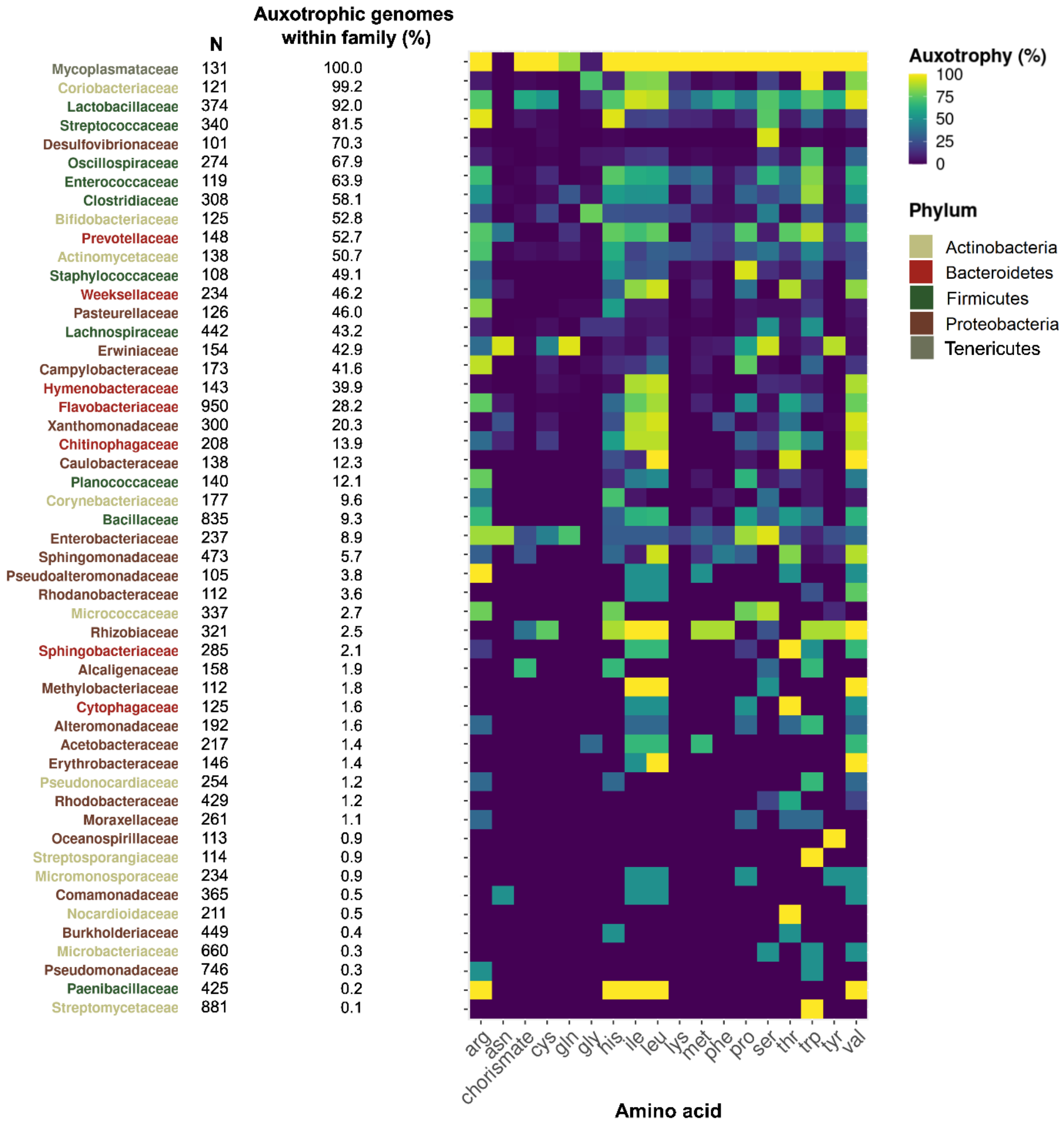

Supplementary Figure 4. Relationships between general genomic features, genome origin, and amino acid auxotrophy in bacteria. A. Genome size of bacterial taxa represented by assembled genomes (MAGs/SAGs) and genomes obtained from bacterial isolates. B. Predicted minimal doubling time in genomes from bacterial taxa represented by assembled genomes (MAGs/SAGs) and genomes obtained from bacterial isolates. C. Correlation between genome size and auxotrophy. Only genomes with more than 95% completeness in the Genome Taxonomy Database (GTDB) are included (N = 26,277 genomes). The correlation coefficients and statistical significance of Pearson's correlation tests are shown on the graph in panel C. Statistical significance on panels A and B was obtained using a Welch two-sample two-sided t-test and a Mann-Whitney U test, respectively. The minimal doubling time was estimated based on the predictive framework of gRodon[1].

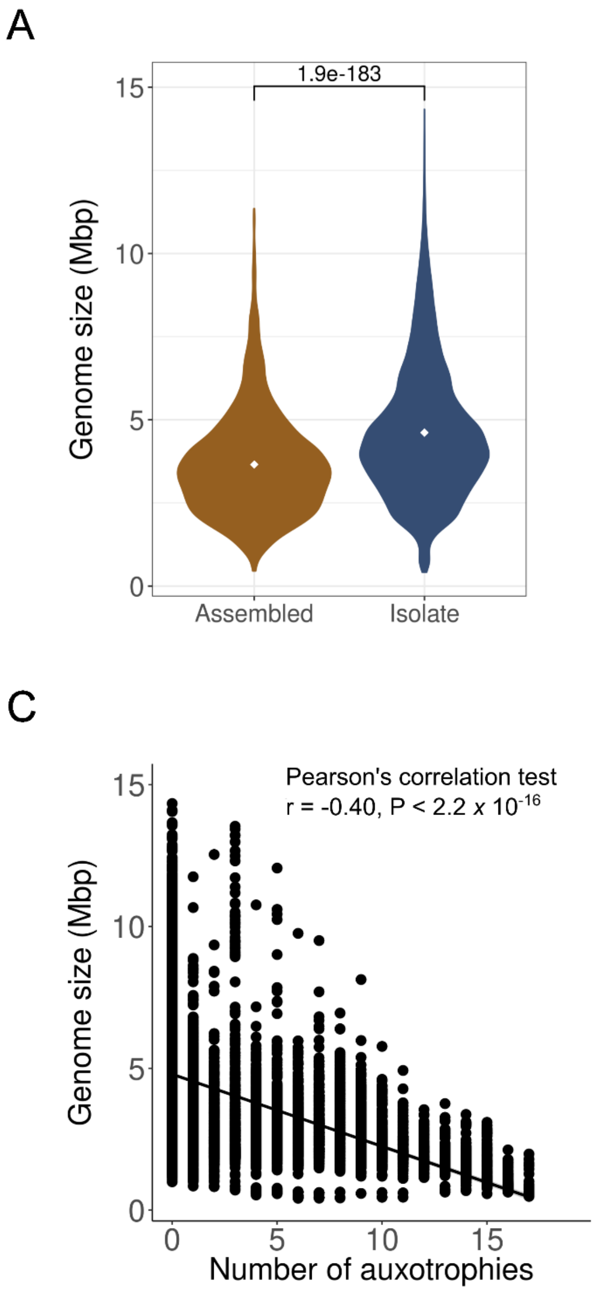

Supplementary Figure 5. Proportion of genomes derived from environmental metagenomes (MAGs) or single cells (SAGs) (Assembled) and genomes obtained from bacterial isolates (Isolate) across the predominant bacterial phyla. The x-axis is sorted by increasing number of amino acid auxotrophies in a given phylum. Numbers on the plot indicate the total number of genomes derived from MAGs/SAGs (upper numbers) and bacterial isolates (lower numbers) within each phylum. Only genomes with more than 95% completeness in the Genome Taxonomy Database (GTDB) are included.

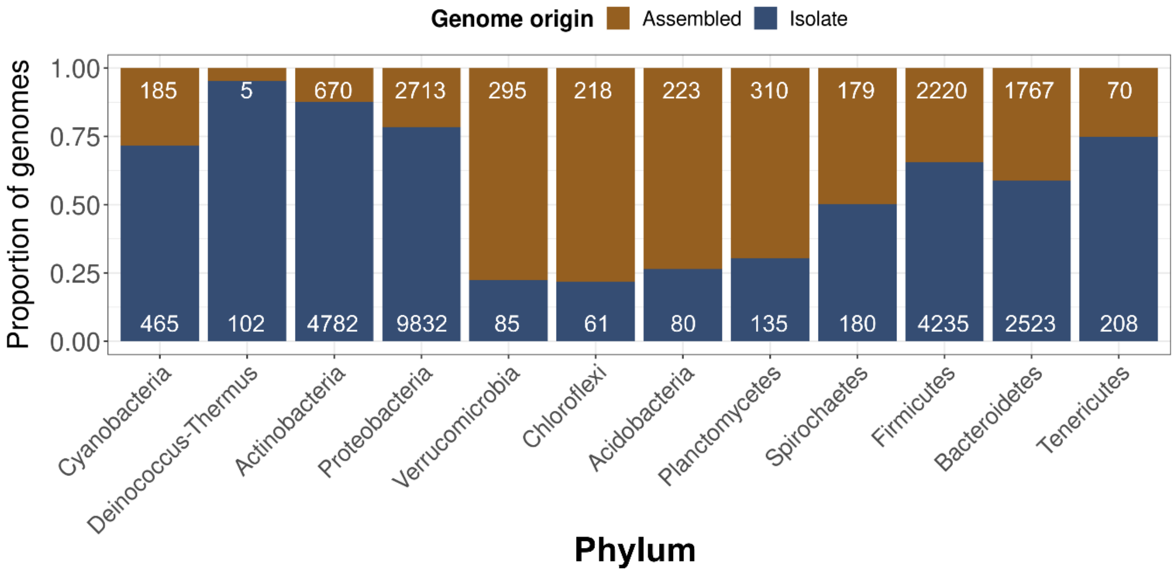

Supplementary Figure 6. Relationships between amino acid auxotrophy and the metabolic cost of amino acid biosynthesis in bacteria. A. Correlation between the biosynthesis cost of 16 amino acids and the proportion of bacterial taxa that are auxotrophic for those amino acids (based on the analysis of 26,277 genomes). B. Correlation between the biosynthesis cost of 17 amino acids and the proportion of auxotrophic taxa for those amino acids in each of the predominant bacterial phyla. The correlation coefficients and statistical significance of Pearson's correlation tests are shown on the graphs. Amino acid biosynthesis costs were obtained from Akashi and Gojobori[2].

A

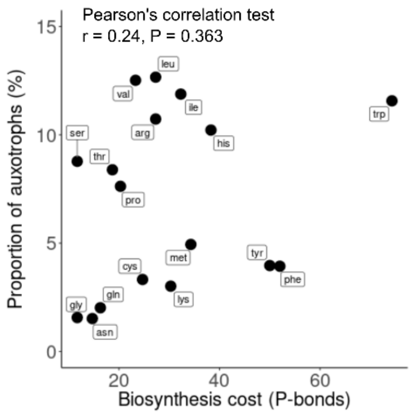

B

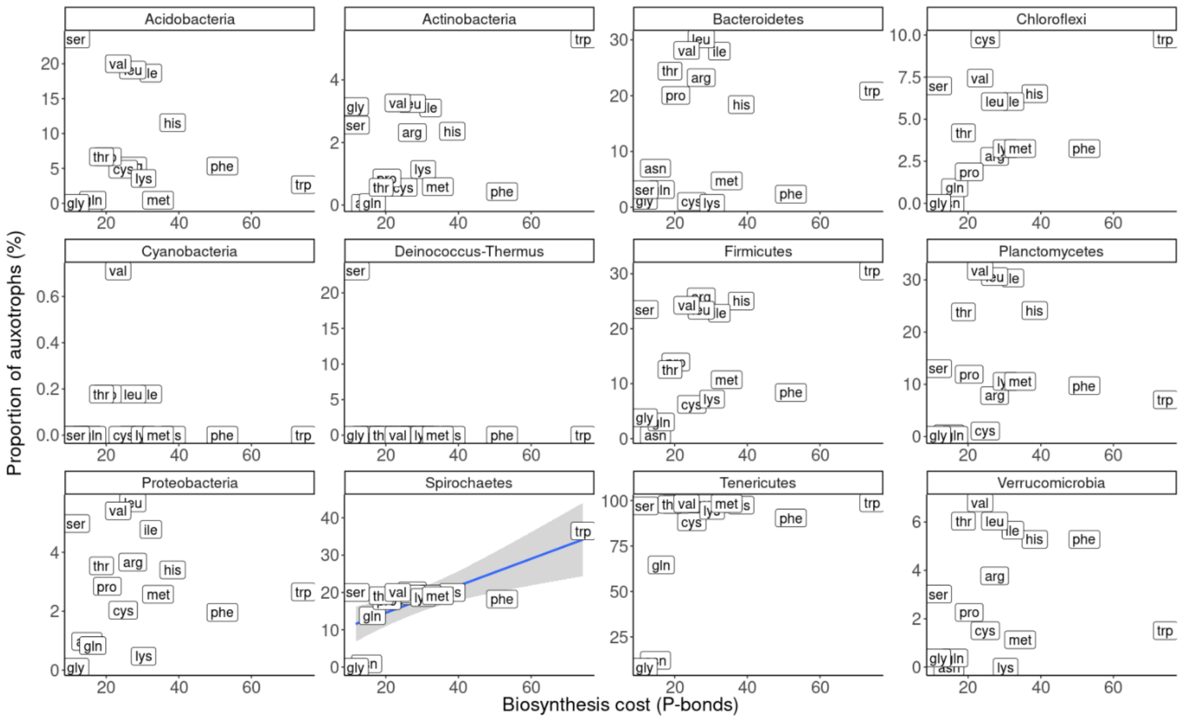

Supplementary Figure 7. Amino acid auxotrophies in the α-, β-, and γ-Proteobacteria across habitats. Numbers in brackets show the number of genomes recovered from each habitat. The mean number of amino acid auxotrophies of representative bacterial taxa in each habitat is shown as red diamonds in the main panel, and as horizontal bars in the top subpanel. Letters on the top subpanel indicate statistical differences ($p < 0.05$) between habitats based on Mann-Whitney U tests with Bonferroni-corrected p-values.

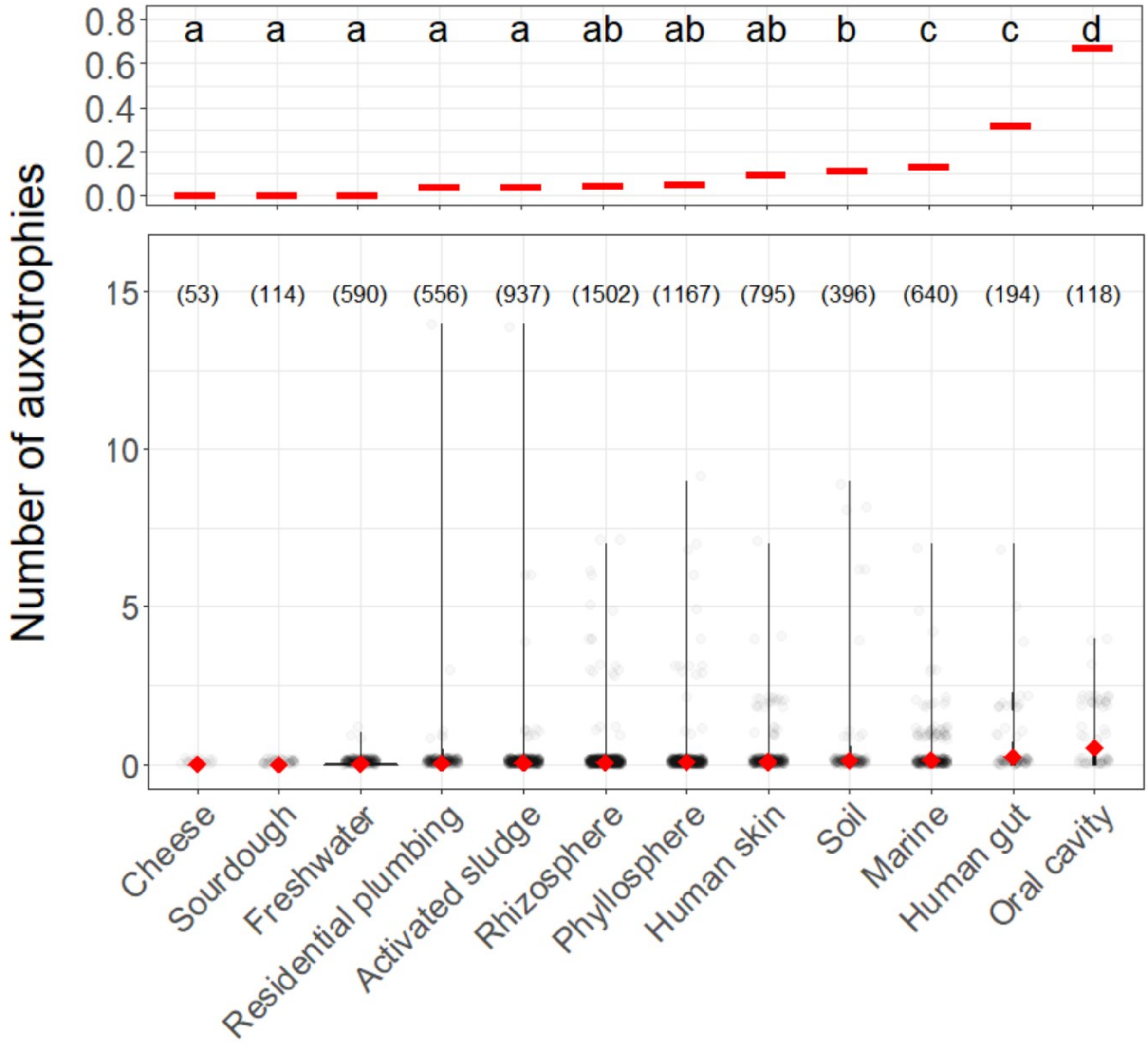

Supplementary Figure 8. Proportion of genomes derived from assembled metagenomes (MAGs/SAGs) and genomes obtained from bacterial isolates in representative genomes of bacterial taxa found across habitats. The x-axis is sorted by increasing number of amino acid auxotrophies in a given habitat. Numbers on the plot indicate the total number of genomes derived from MAGs/SAGs (upper numbers) and bacterial isolates (lower numbers) within each habitat. Only genomes with more than 95% completeness in the Genome Taxonomy Database (GTDB) are included.

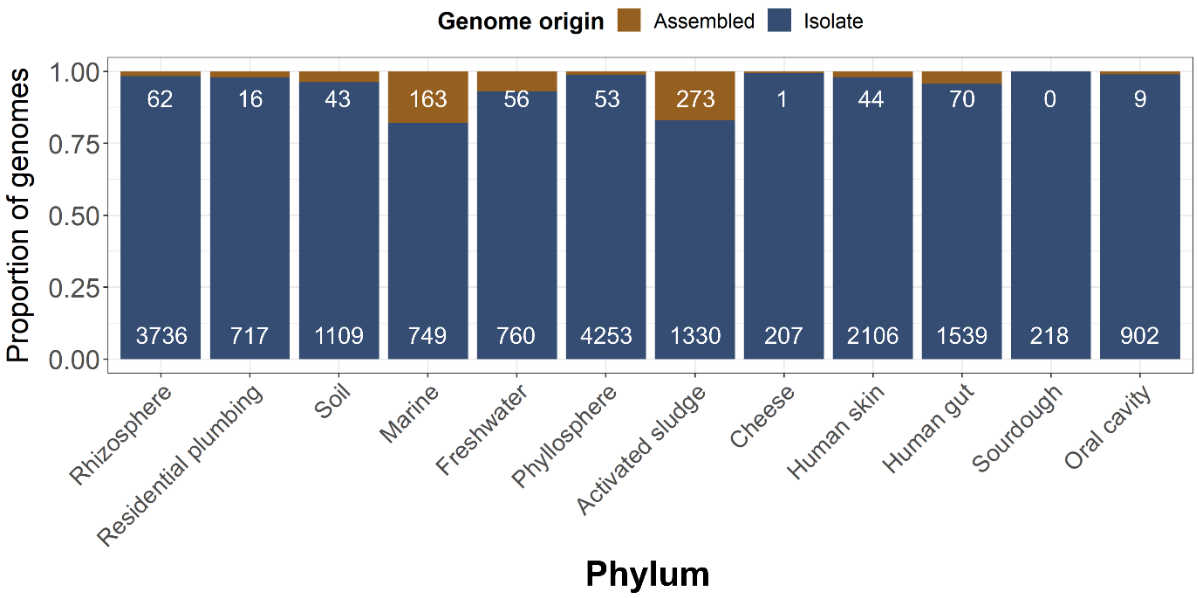

Supplementary Figure 9. Amino acid auxotrophy in the predatory group Bdellovibrionaceae and the putative soil bacterial scavenger *Candidatus* Udaeobacter. The x-axis shows the GenBank genome accession numbers of the taxa.

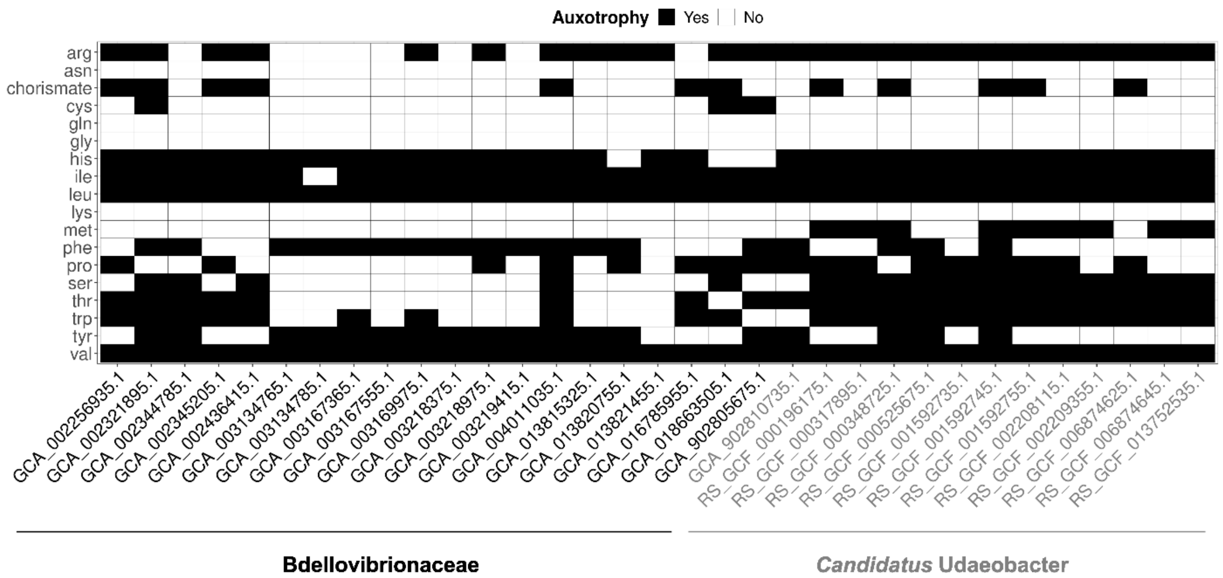

Supplementary Table 1. Genome accessions of 19 bacterial taxa with empirically determined amino acid auxotrophies.

Genome (GenBank)	Strain	Known amino acid auxotrophies	Reference
GCA_000020225.1	*Akkermansia muciniphila* ATCC BAA-835	thr	3
GCA_000013285.1	*Clostridium perfringens* PX7	arg, his, ile, leu, met, phe, thr, trp, tyr, val	4
GCA_004295125.1	*Clostridium scindens* ATCC 37504	trp	5
GCA_001444695.2	*Clostridium sporogenes* PA 3679	arg, ile, leu, phe, trp, tyr, val	6
GCA_000210555.1	*Coprococcus catus* GD/7	trp	7
GCA_009650945.1	*Eikenella corrodens* EI_01	arg, his, lys, pro, ser	8
GCA_000392875.1	*Enterococcus faecalis* V583	arg, his, ile, leu, met, ser, trp, val	9
GCA_000209855.1	*Faecalibacterium prausnitzii* ATCC 27768	trp	7
GCA_000007325.1	*Fusobacterium nucleatum* ATCC 25586	his, lys, ser	10
GCA_003019655.1	*Fusobacterium varium* ATCC 27725	arg, ile, leu, met, val	11
GCA_000008525.1	*Helicobacter pylori* 26695	arg, cys, his, ile, leu, met, phe, pro, val	12
GCA_000742135.1	*Klebsiella pneumoniae* KP11	arg	13
GCA_000409675.1	Lactobacillus *delbrueckii* CRL581	arg, cys, gly, his, ile, leu, phe, ser, trp, tyr, val	14
GCA_000160855.1	*Lactobacillus helveticus* CNRZ 32	arg, his, ile, leu, lys, met, phe, pro, ser, thr, trp, val	15
GCA_000194785.1	*Lactobacillus paracasei* LC2W	arg, ile, leu, phe, ser, trp, tyr, val	16
GCA_002080125.1	*Moraxella catarrhalis* CCRI-195ME	arg	17
GCA_000012705.1	*Streptococcus agalactiae* A909	arg, his, ile, leu, lys, met, phe, trp, tyr, val	18
GCA_000157955.1	*Subdoligranulum variabile* DSM 15176	trp	7
GCA_001047675.1	*Yersinia aleksiciae* 159	trp	13

Supplementary Table 2. Comparison of the predicted proportions of prototrophic taxa for all amino acids using different thresholds of missing genes in a given biosynthesis pathway. Taxa were considered auxotrophic for a particular amino acid if they were missing at least 30%, 40% or 50% of the genes in the biosynthesis pathway of that amino acid. The proportion of taxa with a single auxotrophy was calculated from the total number of auxotrophic taxa.

Phylum	#Genomes	% prototrophs (30% missing genes)	% single auxotrophy (30 % missing genes)	% prototrophs (40% missing genes)	% single auxotrophy (40 % missing genes)	% prototrophs (50% missing genes)	% single auxotrophy (50 % missing genes)
Cyanobacteria	563	52.9	95.1	99.1	80.0	99.8	0
Deinococcus-Thermus	104	7.7	100	76.9	100	76.9	100
Actinobacteria	5088	84.6	47.0	91.4	37.4	91.9	38.2
Proteobacteria	11077	79.8	55.9	89.6	29.0	90.1	30.4
Verrucomicrobia	265	46.4	69.0	84.9	50.0	89.4	35.7
Chloroflexi	215	43.3	52.5	73.0	50.0	75.3	47.2
Acidobacteria	225	9.8	49.8	55.1	43.6	56.4	49.0
Bacteroidetes	3232	35.0	40.4	62.8	10.8	64.1	11.8
Planctomycetes	369	22.8	53.7	62.3	16.6	63.1	15.4
Firmicutes	4674	33.3	33.0	63.0	25.5	54.1	27.1
Spirochaetes	225	29.3	35.8	59.1	35.9	60.4	34.8
Tenericutes	240	0.4	0.4	0.8	0.4	0.8	0.4

Supplementary References

1. Weissman, J. L., Hou, S. & Fuhrman, J. A. Estimating maximal microbial growth rates from cultures, metagenomes, and single cells via codon usage patterns. *Proc. Natl. Acad. Sci. USA* **118**, e2016810118; 10.1073/pnas.2016810118 (2021).
2. Akashi, H. & Gojobori, T. Metabolic efficiency and amino acid composition in the proteomes of *Escherichia coli* and *Bacillus subtilis*. *Proc. Natl. Acad. Sci. USA* **99**, 3695–3700 (2002).
3. Ottman, N. *et al.* Genomescale model and omics analysis of metabolic capacities of *Akkermansia muciniphila* reveal a preferential mucin-degrading lifestyle. *Appl. Environ. Microbiol.* **83**, 1014–1031 (2017).
4. Goldner, S. B., Solberg, M. & Post, L. S. Development of a minimal medium for *Clostridium perfringens* by using an anaerobic chemostat. *Appl. Environ. Microbiol.* **50**, 202–206 (1985).
5. Devendran, S. *et al. Clostridium scindens* ATCC 35704: Integration of nutritional requirements, the complete genome sequence, and global transcriptional responses to bile acids. *Appl. Environ. Microbiol.* **85**, e00052; 10.1128/AEM.00052-19 (2019).
6. Storari, M. *et al.* Genomic approach to studying nutritional requirements of *Clostridium tyrobutyricum* and other Clostridia causing late blowing defects. *Food Microbiol.* **59**, 213–223 (2016).
7. Soto-Martin, E. C. *et al.* Vitamin biosynthesis by human gut butyrate-producing bacteria and cross-feeding in synthetic microbial communities. *MBio* **11**, 1–18 (2020).
8. Keudell, K. C., Robertson, N. L. & Gilles, P. Amino acid requirements of *Eikenella corrodens*. *Microbios* **47**, 73–81 (1986).
9. Murray, B. E. *et al.* Generation of restriction map of *Enterococcus faecalis* OG1 and investigation of growth requirements and regions encoding biosynthetic function. *J. Bacteriol.* **175**, 5216–5223 (1993).
10. Rogers, A. H., Chen, J., Zilm, P. S. & Gully, N. J. The behaviour of *Fusobacterium nucleatum* chemostat-grown in glucose- and amino acid-based chemically defined media. *Anaerobe* **4**, 111–116 (1998).
11. Resmer, K. L. & White, R. L. Metabolic footprinting of the anaerobic bacterium *Fusobacterium varium* using 1H NMR spectroscopy. *Mol. Biosyst.* **7**, 2220–2227 (2011).
12. Testerman, T. L., Conn, P. B., Mobley, H. L. T. & McGee, D. J. Nutritional requirements and antibiotic resistance patterns of *Helicobacter* species in chemically defined media. *J. Clin. Microbiol.* **44**, 1650–1658 (2006).
13. Seif, Y. *et al.* Metabolic and genetic basis for auxotrophies in Gram-negative species. *Proc. Natl. Acad. Sci. USA* **117**, 6264–6273 (2020).
14. Hébert, E. M., Raya, R. R. & De Giori, G. S. Nutritional requirements of *Lactobacillus delbrueckii* subsp. lactis in a chemically defined medium. *Curr. Microbiol.* **49**, 341–345 (2004).
15. Christiansen, J. K. *et al.* Phenotypic and genotypic analysis of amino acid auxotrophy in *Lactobacillus helveticus* CNRZ 32. *Appl. Environ. Microbiol.* **74**, 416–423 (2008).
16. Xu, N., Liu, J., Ai, L. & Liu, L. Reconstruction and analysis of the genome-scale metabolic model of *Lactobacillus casei* LC2W. *Gene* **554**, 140–147 (2015).

17. Jones, M. M. *et al.* Role of the oligopeptide permease ABC transporter of *Moraxella catarrhalis* in nutrient acquisition and persistence in the respiratory tract. *Infect. Immun.* **82**, 4758–4766 (2014).
18. Milligan, T. W., Doran, T. I., Straus, D. C. & Mattingly, S. J. Growth and amino acid requirements of various strains of Group B Streptococci. *J. Clin. Microbiol.* **7**, 28–33 (1978).
