# Supplementary material for: Taxonomic and environmental distribution of bacterial amino acid auxotrophies
Source: Nat Commun. 2023 Nov 22;14:7608. doi: 10.1038/s41467-023-43435-4 (PMC10665431; doi:10.1038/s41467-023-43435-4)
Supplement: Supplementary file 3 — Description of Additional Supplementary Files [file 41467_2023_43435_MOESM3_ESM.pdf]

Description of Additional Supplementary Files:

Supplementary Data 1: Genome accessions and taxonomic affiliation of 171 bacterial taxa that grow in minimal media (i.e. amino acid prototrophs).
