# Supplementary material for: Taxonomic and environmental distribution of bacterial amino acid auxotrophies
Source: Nat Commun. 2023 Nov 22;14:7608. doi: 10.1038/s41467-023-43435-4 (PMC10665431; doi:10.1038/s41467-023-43435-4)
Supplement: Supplementary file 5 — Reporting Summary [file 41467_2023_43435_MOESM5_ESM.pdf]

nature portfolio

Corresponding author(s): Josep Ramoneda and Noah Fierer

Last updated by author(s): Oct 20, 2023

Reporting Summary

Nature Portfolio wishes to improve the reproducibility of the work that we publish. This form provides structure for consistency and transparency in reporting. For further information on Nature Portfolio policies, see our Editorial Policies and the Editorial Policy Checklist.

Statistics

For all statistical analyses, confirm that the following items are present in the figure legend, table legend, main text, or Methods section.

n/a	Confirmed	
☐	☒	The exact sample size (*n*) for each experimental group/condition, given as a discrete number and unit of measurement
☐	☒	A statement on whether measurements were taken from distinct samples or whether the same sample was measured repeatedly
☐	☒	The statistical test(s) used AND whether they are one- or two-sided<br>*Only common tests should be described solely by name; describe more complex techniques in the Methods section.*
☐	☒	A description of all covariates tested
☐	☒	A description of any assumptions or corrections, such as tests of normality and adjustment for multiple comparisons
☐	☒	A full description of the statistical parameters including central tendency (e.g. means) or other basic estimates (e.g. regression coefficient) AND variation (e.g. standard deviation) or associated estimates of uncertainty (e.g. confidence intervals)
☐	☒	For null hypothesis testing, the test statistic (e.g. *F*, *t*, *r*) with confidence intervals, effect sizes, degrees of freedom and *P* value noted<br>*Give P values as exact values whenever suitable.*
☒	☐	For Bayesian analysis, information on the choice of priors and Markov chain Monte Carlo settings
☒	☐	For hierarchical and complex designs, identification of the appropriate level for tests and full reporting of outcomes
☐	☒	Estimates of effect sizes (e.g. Cohen's *d*, Pearson's *r*), indicating how they were calculated

Our web collection on statistics for biologists contains articles on many of the points above.

Software and code

Policy information about availability of computer code

Data collection	No software was used to collect the data as this was available for direct download from open websites..
Data analysis	All code necessary to replicate this work has been uploaded on Figshare (https://doi.org/10.6084/m9.figshare.24101742.v1), and the specifics of the softwares for data analysis have been included in the Methods section. Here a description of these softwares/packages: Genome completeness, CheckM (v1.1.6). Annotation of metabolic pathways, GapMind (adapted code available in Figshare and specifics in doi.org/10.1128/msystems.00291-20). DNA sequence processing, DADA2 pipeline (v1.14.1). Microbial community analysis, phyloseq R package (v1.38.0). Genome annotation into orthologous genes, eggNOG-mapper (v2). Finding representative genomes of ASVs based on 16S rRNA gene sequence similarity, vsearch (v2.23.0). General statistical analysis, R (v4.1.3).

For manuscripts utilizing custom algorithms or software that are central to the research but not yet described in published literature, software must be made available to editors and reviewers. We strongly encourage code deposition in a community repository (e.g. GitHub). See the Nature Portfolio guidelines for submitting code & software for further information.

Data

Policy information about availability of data

All manuscripts must include a data availability statement. This statement should provide the following information, where applicable:

- Accession codes, unique identifiers, or web links for publicly available datasets
- A description of any restrictions on data availability
- For clinical datasets or third party data, please ensure that the statement adheres to our policy

All sequence data analyzed for this study had already been deposited in open repositories and can be accessed through the specific works cited in this work. The source data to reproduce the findings of this study has been made publicly available on Figshare (https://doi.org/10.6084/m9.figshare.24101742.v1). The genome data included in this study can be found in the Genome Taxonomy Database (GTDB, https://data.gtdb.ecogenomic.org/releases/release207/207.0/). Information on predicted doubling times in bacteria can be found in the EGGO database (https://github.com/jlw-ecoevo/eggo). Functional gene annotations were based on the Database of Clusters of Orthologous Genes (COGs, https://www.ncbi.nlm.nih.gov/research/cog).

Research involving human participants, their data, or biological material

Policy information about studies with human participants or human data. See also policy information about sex, gender (identity/presentation), and sexual orientation and race, ethnicity and racism.

Reporting on sex and gender	n/a
Reporting on race, ethnicity, or other socially relevant groupings	n/a
Population characteristics	n/a
Recruitment	n/a
Ethics oversight	n/a

Note that full information on the approval of the study protocol must also be provided in the manuscript.

Field-specific reporting

Please select the one below that is the best fit for your research. If you are not sure, read the appropriate sections before making your selection.

☐ Life sciences ☐ Behavioural & social sciences ☒ Ecological, evolutionary & environmental sciences

For a reference copy of the document with all sections, see nature.com/documents/nr-reporting-summary-flat.pdf

Ecological, evolutionary & environmental sciences study design

All studies must disclose on these points even when the disclosure is negative.

Study description	In this study we comprehensively evaluate the prevalence of amino acid auxotrophy and associated life history strategies across the bacterial tree of life. We predicted the amino acid biosynthesis capabilities of 26,277 genomes representing the 12 most common bacterial phyla. We additionally ran specific analyses for the phyla Bacteroidetes (3,232 genomes) and Firmicutes (4,674 genomes) on the specific life history strategies that characterize amino acid auxotrophs. Also, we ran a cross-habitat analysis of auxotrophy that involved 13,523 genomes from across 3,813 samples from microbial communities found across 12 distinct habitats.
Research sample	Our research samples are 1) full genomes publicly available from the Genome Taxonomy Database (GTDB, https://data.gtdb.ecogenomic.org/releases/release207/207.0/), and 2) samples from microbial communities where the 16S rRNA gene was sequenced to identify the bacterial diversity found in those samples. These samples came from published studies across soils, rhizospheres, phyllospheres, marine environments, freshwater, plumbing systems, gut, oral, and skin microbiomes, cheese, and sourdough. The rationale behind using full genomes for this study is that metabolic predictions can only be performed on complete genomic information representing individual bacterial taxa, and the choice of diverse 16S rRNA sequencing datasets ensured we covered the main bacterial habitats and allowed us to obtain representative genomes where metabolic predictions could be performed.
Sampling strategy	Our analysis started from a set of over 62,000 genomes that were filtered for quality and completeness, yielding a total of 26,277 genomes for analysis. These genomes cover the most predominant phyla in the bacterial tree of life, and is to our knowledge the largest sample size used to evaluate the prevalence of amino acid auxotrophy in bacteria to date.
Data collection	The first author collected the data by downloading the genome sequences from the Genome Taxonomy Database (GTDB) and downloading the microbial community sequence information from the Sequence Read Archive (SRA) from the NCBI.
Timing and spatial scale	All data was collected within a period of 1 month.

Data exclusions	We excluded genomes and microbial community samples from the study that did not meet DNA quality standards of completeness, minimum sequencing depth, chimerism, or genome assembly parameters.
Reproducibility	All the code to reproduce this study has been uploaded to Figshare (https://doi.org/10.6084/m9.figshare.24101742.v1).
Randomization	Randomization was not necessary because this study is based on the analysis of all quality genomes belonging to major bacterial phyla, meaning there was no subsampling that would require randomizing sample choice, experimental designs, or analyses.
Blinding	Not applicable as the nature of data collection did not give room for collection biases.

Did the study involve field work? ☐ Yes ☒ No

Reporting for specific materials, systems and methods

We require information from authors about some types of materials, experimental systems and methods used in many studies. Here, indicate whether each material, system or method listed is relevant to your study. If you are not sure if a list item applies to your research, read the appropriate section before selecting a response.

Materials & experimental systems

n/a	Involved in the study	
☒	☐	Antibodies
☒	☐	Eukaryotic cell lines
☒	☐	Palaeontology and archaeology
☒	☐	Animals and other organisms
☒	☐	Clinical data
☒	☐	Dual use research of concern
☒	☐	Plants

Methods

n/a	Involved in the study	
☒	☐	ChIP-seq
☒	☐	Flow cytometry
☒	☐	MRI-based neuroimaging
